# Feasibility and Safety of a Combined Metabolic Strategy in Glioblastoma Multiforme: A Prospective Case Series

**DOI:** 10.1155/2022/4496734

**Published:** 2022-10-14

**Authors:** M. C. L. Phillips, J. Leyden, E. J. McManus, D. G. Lowyim, F. Ziad, B. G. Moon, N. A. B. Haji Mohd Yasin, A. Tan, Z. Thotathil, M. B. Jameson

**Affiliations:** ^1^Department of Neurology, Waikato Hospital, Hamilton, New Zealand; ^2^Calvary Adelaide Hospital, Adelaide, Australia; ^3^Waikato Clinical Campus, University of Auckland, Hamilton, New Zealand; ^4^Department of Pathology, Waikato Hospital, Hamilton, New Zealand; ^5^Department of Radiology, Waikato Hospital, Hamilton, New Zealand; ^6^Department of Radiation Oncology, Waikato Hospital, Hamilton, New Zealand; ^7^Department of Oncology, Waikato Hospital, Hamilton, New Zealand

## Abstract

**Background:**

Glioblastoma multiforme (GBM) may be susceptible to metabolic strategies such as fasting and ketogenic diets, which lower blood glucose and elevate ketones. Combining these two strategies may be an ideal approach for sustaining a potentially therapeutic glucose ketone index (GKI). In this prospective case series, we observed whether a combined metabolic strategy was feasible, safe, and capable of sustaining a GKI <6 in patients with GBM.

**Methods:**

We provided recommendations and guidelines to 10 GBM patients at various stages of tumour progression and treatment that enabled them to complete a 5–7-day fast every 1–2 months combined with a modified ketogenic diet during the intervening weeks. Patients monitored their blood glucose and ketone levels and body weight. Adverse effects were assessed.

**Results:**

Patients completed a mean of 161 ± 74 days of the combined metabolic strategy, with 34 ± 18 (21%) days of prolonged fasting (mean fast duration: 6.0 ± 1.4 days) and 127 ± 59 (79%) days on the ketogenic diet. The mean GKI for all 10 patients was 3.22 (1.28 during the fasts, 5.10 during the ketogenic diet). Body weight decreased by 8.4 ± 6.9 kg (11.2% decrease in baseline weight). The most common adverse effects attributed to the fasts and ketogenic diet were fatigue, irritability, and feeling lightheaded. The metabolic strategy did not interfere with standard oncological treatments.

**Conclusion:**

This is the first study to observe the feasibility and safety of repeated, prolonged fasting combined with a modified ketogenic diet in patients with GBM. Using minimal support, patients maintained the combined metabolic strategy for 5–6 months while sustaining a potentially therapeutic mean GKI of 3.22. Weight loss was considerable. Adverse effects attributed to the metabolic strategy were mild, and it did not interfere with standard oncological treatments. Study Registration: This study is registered on the Australia New Zealand Clinical Trials Registry, number ACTRN12620001310954. The study was registered on 4 December 2020.

## 1. Introduction

Nearly a century has passed since Warburg proposed that cancer arises from irreversible injury to cell respiration followed by compensation through fermentation energy [1]. Reprogrammed energy metabolism is now recognized as a hallmark of cancer [2], with dramatically increased glucose uptake observed as a near-universal feature of malignant tumours [3]. Tumours may therefore be susceptible to metabolic strategies that target their abnormal metabolism [4]. One such strategy is fasting, a voluntary abstinence from food and drink that permits water, calorie-free fluids, or limited calorie-restricted meals for specified periods of time [5]. Another strategy is a ketogenic diet, a high-fat, adequate-protein, low-carbohydrate diet with daily carbohydrate intake kept below 50 g daily [6]. Both strategies restrict glucose availability while generating fat-derived ketones, which tumours are unable to effectively metabolize for energy [4].

The most common primary malignant brain tumour in adults is glioblastoma multiforme (GBM) [7], which is associated with a median survival of 12–15 months despite optimal treatment [8, 9]. In theory, metabolic strategies may be therapeutically beneficial in GBM by lowering the blood glucose-to-ketone (beta-hydroxybutyrate) ratio, which some investigators have termed the glucose ketone index (GKI) [10]. The GKI compares the relative levels of the primary fermentable fuel (glucose) and the nonfermentable fuel (ketones) using a simple equation: GKI = [glucose (mg/dL) / 18.016 (g^∗^dL/mol)] / [ketones (mmol)] [10]. Lowering the GKI evokes differential stress resistance, in which normal cells display enhanced resistance to multiple stressors (including radiation and chemotherapy) compared with cancer cells, and differential stress sensitization, in which cancer cells display enhanced sensitivity to multiple stressors compared with normal cells [11, 12]. The ideal therapeutic GKI (if one exists) has not been clearly defined in humans, although animal data indicate a GKI <6 is associated with reduced brain tumour volumes and increased survival, particularly in the setting of concurrent radiation or chemotherapy [10].

Excluding case reports, few human studies have examined fasting or ketogenic diets in conjunction with standard oncological treatments in patients with GBM. Only one clinical trial has examined the impact of prolonged fasting (2 days or longer) combined with a ketogenic diet, which was limited to a 9-day treatment period incorporating a single 3-day fast [13]. Several studies have examined the impact of ketogenic diets in GBM patients [14–22], but many of these studies involved a short diet treatment period (≤12 weeks) and only two of them recorded blood glucose and ketone data. In the first such study, 11 patients were enrolled in a 14-week ketogenic diet, with 9 patients achieving a mean GKI <6 and 6 patients completing the diet treatment period [22]. In the second study, 8 patients were enrolled into a 6-month ketogenic diet, with 5 patients achieving a mean GKI <6 and the same number completing the diet treatment period [16].

Given the obvious challenges associated with sustaining a potentially therapeutic GKI for several months or longer, a metabolic strategy consisting of repeated, prolonged fasting combined with a ketogenic diet may be a more viable approach, but this has not been investigated. On this background, we observed whether a combined metabolic strategy was feasible, safe, and capable of sustaining a GKI <6 in patients with GBM.

## 2. Methods

### 2.1. Study Design

This was a prospective case series based out of Waikato Hospital, a tertiary hospital in Hamilton, New Zealand. The study started out as a case report, which the Waikato Hospital Research Office prospectively advised did not require ethics review. However, when additional patients with GBM voiced an interest in using fasting and/or a ketogenic diet as a potential treatment strategy to their neurologist or oncologist, a scope of review form was submitted to the Health and Disability Ethics Committee of New Zealand to commence a case series, which formally waived the requirement for ethics review. The study was then converted into a case series.

### 2.2. Patients

Patients who expressed a desire to explore additional treatment options for GBM were referred by their neurologist or oncologist to the lead investigator for further information on a combined metabolic strategy. Eligibility criteria required that patients were aged 18 years or older, had a histologically confirmed diagnosis of GBM, and did not have a coexisting medical or psychiatric disorder that would impede their ability to follow the fasts or ketogenic diet (the histological criterion was waived for one patient whose tumour could not be biopsied as it was restricted to the thalamus, pons, and midbrain, but based on clinical and imaging features was diagnosed and treated as GBM). In total, 10 patients were referred between March and December 2020, all of whom were included in the study. Written informed consent was obtained from all patients with respect to the use of their hospital records and any data recorded during the metabolic strategy.

### 2.3. Recommendations and Guidance

Patients and their spouses attended a 1-hour visit with the lead investigator, who provided recommendations and guidelines on how to safely conduct a metabolic strategy consisting of a 5–7-day fast (allowing only water, salt, tea, and coffee) every 1-2 months combined with a modified ketogenic diet (consisting mainly of green vegetables, meats, eggs, nuts, seeds, creams, and natural oils) during the intervening weeks. Patients were recommended to conduct their first fast 1-2 weeks after commencing the ketogenic diet, with subsequent fasts conducted once per calendar month during a week of the patient's choosing. After obtaining written informed consent, sample recipes were provided for the ketogenic diet, which consisted of an average macronutrient ratio of 60% fat, 30% protein, 5% fibre, and 5% net carbohydrate by weight. Patients were also advised how to obtain, use, and record data from a blood glucose and ketone monitor (CareSens Dual, Pharmaco Diabetes, Auckland, New Zealand). For consistency, patients were recommended to measure their glucose and ketone data at the same time every day (bedtime). The potential risks and benefits of fasting and ketogenic diets were explained, including simple measures on how to avoid common adverse effects. To provide further information throughout the metabolic strategy, the lead investigator delivered a global e-mail once a week to all patients as well as individual e-mail, phone, or video support (Zoom, Zoom Video Communications, San Jose, USA) as needed.

### 2.4. Data Collection

Data regarding patient characteristics and standard oncological treatments prior to commencing the metabolic strategy were obtained from hospital records. Patients self-documented their blood glucose and ketone levels (daily during the fasts and 3 days a week during the ketogenic diet) as well as their body weight. Patients continued the metabolic strategy for as long as they wished and ceased of their own volition. At the end, a study-specific questionnaire was administered to document any adverse effects attributed by the patients or their spouses to the fasts or ketogenic diet.

### 2.5. Data Analysis

Due to the case series design and small sample size, data were analyzed using descriptive statistics. GKIs were calculated using the aforementioned equation [10]. Data are generally presented as mean ± standard deviation unless stated otherwise.

## 3. Results

### 3.1. Baseline Characteristics

Patient demographic, physical, and tumour characteristics at the time of commencing the combined metabolic strategy are shown in Table 1. Out of 10 patients, 2 patients had already experienced ongoing tumour progression after their initial resection, with 1 patient showing both a recurrence of the original tumour as well as biopsy-proven metastases to both cervical lymph nodes.

### 3.2. Standard Oncological Treatments

Standard treatments utilized prior to or during the combined metabolic strategy are summarized in Table 2. Prior to commencing the metabolic strategy, 3 patients had not undergone a resection (2 patients had inoperable tumours involving the thalamus and brainstem, and 1 patient was managed palliatively by their oncologist) and another 2 patients had already undergone a repeat resection due to tumour recurrence. With respect to chemoradiation, 1 patient declined chemoradiation, 1 patient received 1 week of palliative radiation, 3 patients received 3–5 weeks of chemoradiation, and 5 patients received 6 weeks of chemoradiation. Chemoradiation overlapped with the metabolic strategy in 2 patients, only one of whom received a full 6 weeks of chemoradiation. With respect to adjuvant temozolomide chemotherapy, 1 patient declined chemotherapy, 1 patient was managed palliatively without chemotherapy, 3 patients received 3–5 cycles of chemotherapy, and 5 patients received 6 cycles of chemotherapy. Adjuvant temozolomide chemotherapy overlapped with the metabolic strategy in 4 patients, only one of whom received a full 6 cycles. All 10 patients received at least 1-2 weeks of daily dexamethasone prior to or during the metabolic strategy, with 6 patients receiving 3–6 weeks of dexamethasone.

### 3.3. Combined Metabolic Strategy

**Figure 1:**
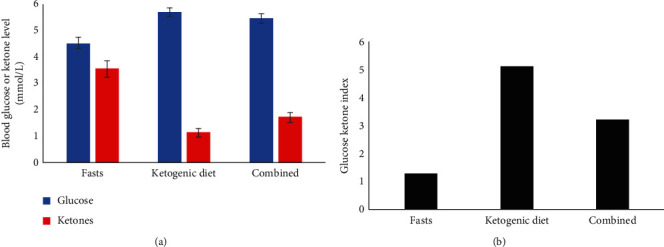
Mean (a) blood glucose and ketone (beta-hydroxybutyrate) levels and (b) glucose ketone indices for all patients during the fasts, ketogenic diet, and combined metabolic strategy (*n* = 10). Error bars indicated standard error.

Metabolic data obtained for the combined metabolic strategy are shown in Table 3 and Figure 1. The mean duration from diagnosis to commencement of the metabolic strategy was 151 ± 128 days. The metabolic strategy lasted a mean of 161 ± 74 days, with 34 ± 18 (21%) days dedicated to fasting and 127 ± 59 (79%) days dedicated to the ketogenic diet. Each patient performed a mean of 5.7 ± 2.7 prolonged fasts, each of which lasted a mean of 6.0 ± 1.4 days. Overall, 9 patients achieved a mean GKI <6 (1 patient achieved a mean GKI of 7.2). For all 10 patients, the mean GKI during the metabolic strategy was 3.22 (1.28 during the fasts, 5.10 during the ketogenic diet). Mean body weight decreased from a baseline of 75.1 ± 13.8 kg to 66.7 ± 14.0 kg, representing a reduction of 8.4 ± 6.9 kg (11.2% decrease in baseline weight). Mean body mass index decreased from a baseline of 25.4 ± 4.3 kg/m^2^ to 22.5 ± 4.2 kg/m^2^, representing a reduction of 2.9 ± 2.3 kg/m^2^ (11.2% decrease in baseline body mass index).

### 3.4. Adverse Effects

Adverse effects that occurred at any time during the combined metabolic strategy are shown in Table 4. The most common adverse effects attributed to both the fasts and ketogenic diet were increased fatigue, increased irritability, and feeling lightheaded. No grade 3 or higher adverse events, classified by the Common Terminology Criteria for Adverse Events (CTCAE) (version 5.0), were attributed by the patient or their spouse to the fasts or ketogenic diet. During the metabolic strategy, 4 patients were admitted to hospital with generalized seizures, all of whom showed radiological evidence of tumour progression at the time. The metabolic strategy did not alter or interfere with the standard treatments in any way, and most patients described subjective improvements in physical activity and mood.

### 3.5. Survival

**Figure 2:**
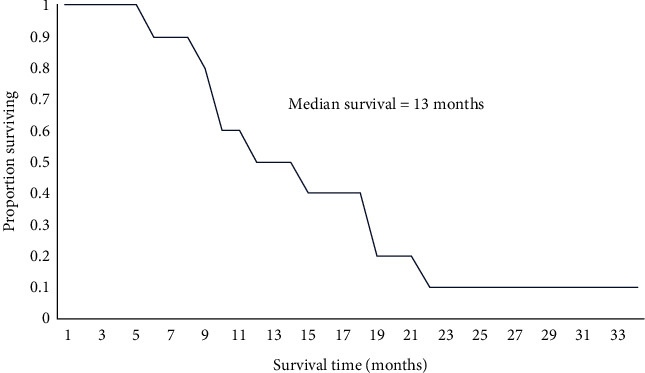
Kaplan–Meier plot of patient survival (*n* = 10).

Survival data are shown in Table 5 and Figure 2. Actual survival times in the study were comparable to expected survival probabilities (calculated using the NRG oncology/RTOG 0525 and 0825 survival calculator at http://cancer4.case.edu/rCalculator/rCalculator.html). At the time of write-up, 9 patients had died, representing a median survival of 13 months for the study. Overall, 7 patients maintained the combined metabolic strategy until they were no longer capable of doing so, which in all cases was within the final month prior to death. The remaining 3 patients ceased the prolonged fasts but continued to restrict their carbohydrate intake.

## 4. Discussion

This is the first study to observe the feasibility and safety of repeated, prolonged fasting combined with a modified ketogenic diet in patients with GBM (or any other advanced cancer). Using minimal support, patients adhered to a combined metabolic strategy for 5-6 months while sustaining a potentially therapeutic mean GKI of 3.22. Weight loss was considerable. Adverse effects attributed to the metabolic strategy were mild, and it did not interfere with standard oncological treatments.

We accepted all referrals into the study, which resulted in a heterogenous group of patients at various stages of tumour progression and prognosis at the time of commencing the combined metabolic strategy. Out of 10 patients, 5 patients had either experienced tumour progression after the initial resection (including 1 patient with extracranial metastases), had an inoperable tumour, or were deemed palliative at the time of diagnosis. The poor prognosis associated with half the patients in this study would have excluded them from many GBM trials.

Most patients had already completed standard oncological treatments prior to commencing the combined metabolic strategy. Chemoradiation overlapped with the metabolic strategy in just 2 patients, only one of whom received a full 6 weeks of chemoradiation. Adjuvant temozolomide chemotherapy overlapped with the metabolic strategy in just 4 patients, only one of whom received a full 6 cycles. In theory, metabolic strategies should be maximally beneficial when administered in conjunction with standard treatments by eliciting differential stress resistance, which increases the resistance of normal cells to radiation and chemotherapy, and differential stress sensitization, which increases the sensitivity of cancer cells to standard treatments [11, 12]. In this study, fewer than half the patients would have achieved these potential benefits, as most were either not able to overlap the metabolic strategy with their standard treatments or did not receive the full standard treatment regimen. Moreover, all 10 patients received dexamethasone during the course of their treatment, which may be problematic from a metabolic standpoint given that corticosteroids elevate blood glucose, the primary fermentable fuel required by cancer cells, which may compromise patient survival [23, 24].

Overall, 9 patients achieved a mean GKI <6 during the 5-6 months of the combined metabolic strategy, with the entire group achieving a mean GKI of 3.22. Given that most prior studies of fasting or ketogenic diets in GBM involved shorter treatment periods [13, 17–20, 22], low adherence [14], or high withdrawals [16, 19, 22], the findings from this study are reasonably encouraging. The repeated, prolonged fasts, which averaged 6 days in length, achieved a mean GKI of 1.28. By comparison, the ketogenic diet achieved a GKI of 5.10, which is only slightly beneath the stated target of a GKI <6. Clearly, despite representing only 21% of days for the metabolic strategy, the fasts lowered the GKI to a much greater extent than would have been achieved by the diet alone. Since a lower GKI theoretically equates to greater degrees of differential stress resistance and sensitization leading to a maximally hostile cell environment for cancer cells, this finding indicates a combined approach involving prolonged fasting and a ketogenic diet may be helpful in achieving a therapeutic GKI, particularly if the fasts are timed in conjunction with radiation or chemotherapy.

Patients lost 11.2% of their baseline body weight during the combined metabolic strategy. This degree of weight loss represents a CTCAE grade 2 adverse event, although no patient required additional nutritional support and the mean body mass index by the end of the metabolic strategy was actually in the ideal range of 22-23 kg/m^2^. Unintentional weight loss is concerning in cancer patients as it can precipitate malnutrition, sarcopenia, and cachexia [25]. However, intentional weight loss is associated with a lowered risk of developing many types of cancer [26–28]. When done properly, both fasting and ketogenic diets can be performed while meeting the patient's nutritional needs and preserving lean mass, with ketogenic diets potentially inducing weight gain (not loss) in cachectic cancer patients [5, 29, 30]. Moreover, intentional weight loss could be perceived as advantageous in cancer patients given that it indicates maximal metabolic pressure being exerted upon cancer cells. In accordance with this possibility, preliminary reports of fasting and ketogenic diet strategies in patients with advanced tumours, including GBM, have shown that weight loss in the range of 13–28% can be associated with highly positive therapeutic outcomes [31–34].

Adverse effects attributed by the patients or their spouses to the fasts or ketogenic diet were mild, with the most common being mildly increased fatigue, irritability, and feeling lightheaded. No adverse events of CTCAE grade 3 or higher occurred. Although 4 patients were admitted to hospital for generalized seizures during the combined metabolic strategy, these seizures all occurred in the context of tumour progression. Importantly, the combined metabolic strategy did not alter or interfere with the standard oncological treatments in any way. Moreover, despite the mild fatigue, most patients experienced beneficial effects in physical function and mood, both of which have been described in previous studies [29, 35].

Recently, the application of a Bayesian analysis to the collective evidence from animal and human studies demonstrated that metabolic strategies may be associated with a survival-prolonging effect in high-grade glioma patients, particularly when combined with other treatment modalities [36]. In this study, the median survival was 13 months, which is comparable with the median survival of 12–15 months reported for GBM patients undergoing the full standard radiation and chemotherapy regimen [8, 9]. It also compares well with the median survival of 12 months reported by hospitals at which patients received multimodal (but not necessarily optimal) treatment, including our own hospital [37, 38]. Moreover, in our cohort, only 5 patients had confirmed methylated GBM and only 5 patients received the full standard treatment regimen. Unmethylated GBM is associated with a median survival of only 12.7 months in patients receiving the full standard treatment regimen [39] and only 10 months in elderly patients receiving a 3-week course of chemoradiation [40]. Given the poor prognosis of many of our patients at the time of commencing the combined metabolic strategy, lack of overlap of the metabolic strategy with standard treatments in most patients, and corticosteroid exposure in all patients, the median survival observed in this study could be interpreted as somewhat encouraging (that being said, it is not possible to make robust conclusions about survival in a case series, particularly one involving small patient numbers).

This study had some strengths. First, we accepted all referred patients, which mitigated selection bias. Second, the combined metabolic strategy was implemented using minimal recommendations and guidelines. It was also inexpensive, imposing no major costs on the patients (the ketogenic diet imposed a slight increase in the weekly cost of food, but the fasts levelled out the costs) or the investigators (no funding was required to conduct the study).

This study also bears several important limitations. First, we utilized a case series design, which is prone to bias and unable to make definitive conclusions. Second, the sample size was very small at only 10 patients. Third, patient heterogeneity with respect to tumour progression, prognosis, and treatment regimens combined with the universal application of corticosteroids makes it difficult for our findings to be extrapolated to many other patients with GBM.

In conclusion, our observations indicate a longer-term (5-6 month) metabolic strategy combining repeated, prolonged fasting with a modified ketogenic diet appears to be feasible, safe, and capable of sustaining a potentially therapeutic GKI <6 in patients with GBM. Given the limitations of this study, our observations should be considered exploratory. Well-designed clinical trials will be required to determine whether a combined metabolic strategy is therapeutically effective in GBM, particularly in conjunction with standard oncological treatment.

## Figures and Tables

**Table 1:** Patient demographic, physical, and tumour characteristics at the time of commencing the combined metabolic strategy.

Characteristic	Patients (*n* = 10)
Demographic	
Age (years)	58.0 +/−11.9 (range, 40–74)
Gender (male)	7 (70%)
Ethnicity	
European	9 (90%)
Asian	1 (10%)
Previous cancer diagnosis	
Breast cancer	2 (20%)
Fibromyxoid sarcoma	1 (10%)
Physical	
Body weight (kg)	75.1 +/−13.8
Body mass index	25.4 +/−4.3
Function (ECOG performance status score)	
0	3 (30%)
1	5 (50%)
2	2 (20%)
Tumour	
Location (bulk of tumour)	
Temporal	4
Frontal	2
Thalamus and brainstem	2
Parietal	1
Occipital	1
Growth status	
Relatively recent diagnosis (serial imaging not yet obtained)	5 (50%)
No progression (no tumour recurrence postresection)	3 (30%)
Progression (tumour recurrence postresection)	2 (10%)
MGMT promoter methylation status	
Unmethylated	3 (30%)
Methylated	5 (50%)
Unknown	2 (20%)
IDH 1 mutation status	
Negative	9 (90%)
Positive	0
Unknown	1 (10%)

^*∗*^Except for % variables, values are presented as mean +/−standard deviation. ^*∗*^ ECOG—eastern cooperative oncology group; MGMT—O6-methylguanine-DNA-methyltransferase; IDH 1—isocitrate dehydrogenase 1.

**Table 2:** Standard treatments utilized prior to or during the combined metabolic strategy.

Treatment	Patients (*n* = 10)
Surgery	
No biopsy or resection (technically impossible)	1 (10%)
Biopsy but no resection (inoperable or palliative)	2 (20%)
Partial resection	6 (60%)
Total resection	1 (10%)
Repeat resection	
Prior to combined metabolic strategy	2 (20%)
During combined metabolic strategy	2 (20%)
Chemoradiation	
No chemoradiation	1 (10%)
1 week of palliative radiation	1 (10%)
3–5 weeks of chemoradiation	
Completed prior to combined metabolic strategy	2 (20%)
Overlapped with combined metabolic strategy	1 (10%)
6 weeks of chemoradiation	
Completed prior to combined metabolic strategy	4 (40%)
Overlapped with combined metabolic strategy	1 (10%)
Mean total radiation dose (Gy)	45.3 +/−19.8
Adjuvant chemotherapy	
No chemotherapy	2 (20%)
3–5 cycles of temozolomide chemotherapy	
Completed prior to combined metabolic strategy	0
Overlapped with combined metabolic strategy	3 (30%)
6 cycles of temozolomide chemotherapy	
Completed prior to combined metabolic strategy	4 (40%)
Overlapped with combined metabolic strategy	1 (10%)
Post-temozolomide chemotherapy (bevacizumab/irinotecan)	2 (20%)
Dexamethasone	
No dexamethasone	0
Daily for 1-2 weeks	4 (40%)
Daily for 3-4 weeks	4 (40%)
Daily for 5-6 weeks	2 (20%)

^*∗*^Except for % variables, values are presented as mean +/−standard deviation.

**Table 3:** Metabolic data for the combined metabolic strategy.

Metabolic data	Patients (*n* = 10)
Time	
Time from diagnosis to commencing combined metabolic strategy (days)	151 +/–128
Time spent undergoing combined metabolic strategy (days)	161 +/–74
Time spent fasting (days)	34 +/–18
Time spent on ketogenic diet (days)	127 +/–59
Number of fasts per patient	5.7 +/–2.7
Length of fasts (days)	6.0 +/–1.4

Blood glucose and ketones (fasts and ketogenic diet combined)	
Blood glucose (mmol/L)	5.44 +/–0.55
Blood ketones (mmol/L)	1.69 +/–0.62
Proportion of patients achieving a glucose ketone index <6	9 (90%)
Mean glucose ketone index, all patients	3.22

Blood glucose and ketones (fasts)	
Blood glucose (mmol/L)	4.50 +/–0.68
Blood ketones (mmol/L)	3.52 +/–0.99
Mean glucose ketone index, all patients	1.28

Blood glucose and ketones (ketogenic diet)	
Blood glucose (mmol/L)	5.66 +/–0.46
Blood ketones (mmol/L)	1.11 +/–0.52
Mean glucose ketone index, all patients	5.10

Change in body weight	
Change in body weight during combined metabolic strategy (kg)	−8.4 +/–6.9
Change in body weight (%)	−11.2%
Change in body mass index during combined metabolic strategy (kg/m^2^)	−2.9 +/–2.3
Change in body mass index (%)	−11.2%

^*∗*^The date of biopsy was taken as the date of diagnosis, except for the patient whose tumour could not be biopsied, in whom the date of the initial MRI scan was taken as the date of diagnosis. ^*∗*^Except for indices and % values, values are presented as mean +/–standard deviation.

**Table 4:** Adverse effects that occurred at any time during the fasts or ketogenic diet (including the level of certainty that patients and spouses attributed each adverse effect to either strategy).

	Definite	Probable	Possible	Unsure	Total
*Fasting adverse effects*					
Increased fatigue	3	0	2	1	6 (60%)
Increased irritability	3	1	2	0	6 (60%)
Feeling lightheaded	2	0	3	0	5 (50%)
Increased limb or back pain	1	1	0	3	5 (50%)
Excessive hunger	2	0	3	0	5 (50%)
Muscle cramps	1	2	0	0	3 (30%)
Sugar cravings	1	0	1	0	2 (20%)
Constipation	0	2	0	0	2 (20%)
Nausea	1	1	0	0	2 (20%)
Diarrhea	1	0	0	0	1 (10%)
Insomnia	1	0	0	0	1 (10%)
Headaches	1	0	0	0	1 (10%)
Palpitations	0	0	1	0	1 (10%)
Disorientation	1	0	0	0	1 (10%)
Generalized seizure	0	0	0	1	1 (10%)
Food aversions	0	0	0	0	0
Heartburn	0	0	0	0	0
Excessive thirst	0	0	0	0	0
Total	18	7	12	5	42

*Ketogenic diet adverse effects*					
Increased fatigue	2	1	0	2	5 (50%)
Increased irritability	0	2	1	1	4 (40%)
Feeling lightheaded	1	0	3	0	4 (40%)
Sugar cravings	1	0	1	2	4 (40%)
Constipation	1	2	1	0	4 (40%)
Increased limb or back pain	0	0	1	2	3 (30%)
Generalized seizure	0	0	0	3	3 (30%)
Excessive hunger	1	1	0	0	2 (20%)
Nausea	0	1	1	0	2 (20%)
Diarrhea	1	1	0	0	2 (20%)
Food aversions	1	0	1	0	2 (20%)
Insomnia	0	0	0	1	1 (10%)
Headaches	0	0	0	1	1 (10%)
Heartburn	0	0	0	1	1 (10%)
Muscle cramps	0	0	0	0	0
Palpitations	0	0	0	0	0
Disorientation	0	0	0	0	0
Excessive thirst	0	0	0	0	0
Total	8	8	9	13	38

**Table 5:** Actual survival times and expected survival probabilities.

Patient	Actual survival (months)	Expected 6-month survival probability	Expected 12-month survival probability	Expected 24-month survival probability
1	11.2	0.75	0.33	0.10
2	33.0 (ongoing)	0.94	0.81	0.54
3	5.2	0.80	0.42	0.10
4	18.0	0.92	0.75	0.44
5	18.6	0.91	0.71	0.36
6	9.5	0.93	0.77	0.46
7	14.8	0.87	0.60	0.23
8	9.4	0.68	0.23	0.10
9	21.6	0.86	0.56	0.19
10	8.7	0.89	0.65	0.28

^*∗*^Expected survival probabilities were calculated using the NRG oncology/RTOG 0525 and 0825 survival calculator at http://cancer4.case.edu/rCalculator/rCalculator.html.

## Data Availability

The data that support the findings of this study are freely available from the corresponding author upon reasonable request.

## References

[1] Warburg O., Posener K., Negelein E. (1924). Über den stoffwechsel der carcinomzelle. *Biochemische Zeitschrift*.

[2] Hanahan D., Weinberg R. A. (2011). Hallmarks of cancer: the next generation. *Cell*.

[3] Warburg O. (1956). On the origin of cancer cells. *Science*.

[4] Seyfried T. N., Mukherjee P. (2005). Targeting energy metabolism in brain cancer: review and hypothesis. *Nutrition and Metabolism*.

[5] Longo V. D., Di Tano M., Mattson M. P., Guidi N. (2021). Intermittent and periodic fasting, longevity and disease. *Nature Aging*.

[6] O’Neill B. J. (2020). Effect of low-carbohydrate diets on cardiometabolic risk, insulin resistance, and metabolic syndrome. *Current Opinion in Endocrinology Diabetes and Obesity*.

[7] Ostrom Q. T., Patil N., Cioffi G., Waite K., Kruchko C., Barnholtz-Sloan J. S. (2020). CBTRUS statistical report: primary brain and other central nervous system tumors diagnosed in the United States in 2013–2017. *Neuro-Oncology*.

[8] Stupp R., Mason W. P., van den Bent M. J. (2005). Radiotherapy plus concomitant and adjuvant temozolomide for glioblastoma. *New England Journal of Medicine*.

[9] Wen P. Y., Kesari S. (2008). Malignant gliomas in adults. *New England Journal of Medicine*.

[10] Meidenbauer J. J., Mukherjee P., Seyfried T. N. (2015). The glucose ketone index calculator: a simple tool to monitor therapeutic efficacy for metabolic management of brain cancer. *Nutrition and Metabolism*.

[11] de Groot S., Pijl H., van der Hoeven J. J. M., Kroep J. R. (2019). Effects of short-term fasting on cancer treatment. *Journal of Experimental & Clinical Cancer Research*.

[12] Klement R. J. (2018). Fasting, fats, and physics: combining ketogenic and radiation therapy against cancer. *Complementary Medicine Research*.

[13] Voss M., Wagner M., von Mettenheim N. (2020). ERGO2: a prospective, randomized trial of calorie-restricted ketogenic diet and fasting in addition to reirradiation for malignant glioma. *International Journal of Radiation Oncology, Biology, Physics*.

[14] Artzi M., Liberman G., Vaisman N. (2017). Changes in cerebral metabolism during ketogenic diet in patients with primary brain tumors: 1H-MRS study. *Journal of Neuro-Oncology*.

[15] Champ C. E., Palmer J. D., Volek J. S. (2014). Targeting metabolism with a ketogenic diet during the treatment of glioblastoma multiforme. *Journal of Neuro-Oncology*.

[16] Klein P., Tyrlikova I., Zuccoli G., Tyrlik A., Maroon J. C. (2020). Treatment of glioblastoma multiforme with “classic” 4 : 1 ketogenic diet total meal replacement. *Cancer & Metabolism*.

[17] Martin-McGill K. J., Marson A. G., Tudur Smith C., Jenkinson M. D. (2018). The modified ketogenic diet in adults with glioblastoma: an evaluation of feasibility and deliverability within the national health service. *Nutrition and Cancer*.

[18] Rieger J., Bähr O., Maurer G. D. (2014). ERGO: a pilot study of ketogenic diet in recurrent glioblastoma. *International Journal of Oncology*.

[19] Santos J. G., Da Cruz W. M. S., Schönthal A. H. (2018). Efficacy of a ketogenic diet with concomitant intranasal perillyl alcohol as a novel strategy for the therapy of recurrent glioblastoma. *Oncology Letters*.

[20] Schwartz K. A., Noel M., Nikolai M., Chang H. T. (2018). Investigating the ketogenic diet as treatment for primary aggressive brain cancer: challenges and lessons learned. *Frontiers in Nutrition*.

[21] Strowd R. E., Cervenka M. C., Henry B. J., Kossoff E. H., Hartman A. L., Blakeley J. O. (2015). Glycemic modulation in neuro-oncology: experience and future directions using a modified Atkins diet for high-grade brain tumors. *Neuro-Oncology Practice*.

[22] van der Louw E. J. T. M., Olieman J. F., van den Bemt P. M. L. A. (2019). Ketogenic diet treatment as adjuvant to standard treatment of glioblastoma multiforme: a feasibility and safety study. *Therapeutic Advances in Medical Oncology*.

[23] Klement R. J., Champ C. E. (2017). Corticosteroids compromise survival in glioblastoma in part through their elevation of blood glucose levels. *Brain*.

[24] Pitter K. L., Tamagno I., Alikhanyan K. (2016). Corticosteroids compromise survival in glioblastoma. *Brain*.

[25] Nencioni A., Caffa I., Cortellino S., Longo V. D. (2018). Fasting and cancer: molecular mechanisms and clinical application. *Nature Reviews Cancer*.

[26] Luo J., Hendryx M., Manson J. E. (2019). Intentional weight loss and obesity-related cancer risk. *JNCI Cancer Spectrum*.

[27] Sjöström L., Gummesson A., Sjöström C. D. (2009). Effects of bariatric surgery on cancer incidence in obese patients in Sweden (Swedish obese subjects study): a prospective, controlled intervention trial. *The Lancet Oncology*.

[28] MacKintosh M. L., Derbyshire A. E., McVey R. J. (2019). The impact of obesity and bariatric surgery on circulating and tissue biomarkers of endometrial cancer risk. *International Journal of Cancer*.

[29] de Cabo R., Mattson M. P. (2019). Effects of intermittent fasting on health, aging, and disease. *New England Journal of Medicine*.

[30] Weber D. D., Aminzadeh-Gohari S., Tulipan J., Catalano L., Feichtinger R. G., Kofler B. (2020). Ketogenic diet in the treatment of cancer-where do we stand?. *Molecular Metabolism*.

[31] Elsakka A. M. A., Bary M. A., Abdelzaher E. (2018). Management of glioblastoma multiforme in a patient treated with ketogenic metabolic therapy and modified standard of care: a 24-month follow-up. *Frontiers in Nutrition*.

[32] Goldhamer A. C., Klaper M., Foorohar A., Myers T. R. (2015). Water-only fasting and an exclusively plant foods diet in the management of stage IIIa, low-grade follicular lymphoma. *BMJ Case Reports*.

[33] Phillips M. C. L., Murtagh D. K. J., Sinha S. K., Moon B. G. (2020). Managing metastatic thymoma with metabolic and medical therapy: a case report. *Frontiers in Oncology*.

[34] Zuccoli G., Marcello N., Pisanello A. (2010). Metabolic management of glioblastoma multiforme using standard therapy together with a restricted ketogenic diet: case report. *Nutrition and Metabolism*.

[35] Williams T. J., Cervenka M. C. (2017). The role for ketogenic diets in epilepsy and status epilepticus in adults. *Clinical Neurophysiology Practice*.

[36] Klement R. J., Bandyopadhyay P. S., Champ C. E., Walach H. (2018). Application of Bayesian evidence synthesis to modelling the effect of ketogenic therapy on survival of high grade glioma patients. *Theoretical Biology and Medical Modelling*.

[37] McManus E. J., Frampton C., Tan A., Phillips M. C. L. (2021). Metabolics risk factors in a New Zealand glioblastoma cohort. *Neuro-Oncology Practice*.

[38] Witthayanuwat S., Pesee M., Supaadirek C., Supakalin N., Thamronganantasakul K., Krusun S. (2018). Survival analysis of glioblastoma multiforme. *Asian Pacific Journal of Cancer Prevention*.

[39] Hegi M. E., Diserens A.-C., Gorlia T. (2005). MGMT gene silencing and benefit from temozolomide in glioblastoma. *New England Journal of Medicine*.

[40] Perry J. R., Laperriere N., O’Callaghan C. J. (2017). Short-course radiation plus temozolomide in elderly patients with glioblastoma. *New England Journal of Medicine*.

